# Optogenetic methods to stimulate gamma motor neuron axons *ex vivo*

**DOI:** 10.1113/EP092359

**Published:** 2025-02-03

**Authors:** Apoorva Karekal, Remie Mandawe, Cameron Chun, Sai Kiran Byri, Danitza Cheline, Serena Ortiz, Shawn Hochman, Katherine A. Wilkinson

**Affiliations:** ^1^ Department of Biological Sciences, One Washington Square San José State University San Jose California USA; ^2^ Department of Cell Biology Emory University School of Medicine Atlanta Georgia USA

**Keywords:** gamma motor neuron, muscle spindle, optogenetics, proprioception, sensorimotor control, somatosensory system

## Abstract

It is challenging to stimulate gamma motor neurons, which are important regulators of muscle spindle afferent function, without also recruiting alpha motor neurons. Here, we test the feasibility of stimulating gamma motor neuron axons using optogenetics in two transgenic mouse lines. We used an *ex vivo* muscle–nerve preparation in adult mice to monitor muscle spindle afferent firing, which should increase in response to gamma motor neuron‐induced lengthening of the sensory region of the muscle spindle. A force transducer measured alpha motor neuron‐mediated twitch contractions. Blue LED light (470 nm; 1–5 mW) was delivered via a light guide to the sciatic nerve. We confirmed that the more slowly conducting gamma motor neurons were recruited first in mice expressing channelrhodopsin 2 in choline acetyltransferase‐positive motor neurons, whereas alpha motor neurons required higher optical intensities, enabling co‐activation of alpha and gamma motor neurons depending on light intensity. However, this approach cannot isolate gamma motor neuron activity completely. Cre‐dependent channelrhodopsin 2 optoactivation using the putative gamma motor neuron marker neuronal PAS domain protein 1 (Npas1) also increased muscle spindle afferent firing rates and caused only small twitch contractions. This provides functional validation that Npas1 is present primarily in gamma motor neurons and can be used to manipulate gamma motor neurons independently. We propose optogenetic stimulation as a promising tool to manipulate gamma motor neuron activity.

## Abstract (highlights)

- **What is the central question of this study?**
  Additional tools are needed to modulate gamma motor neuron function independently to gain a better understanding of the function of the fusimotor system.
- **What is the main finding and its importance?**
  Optogenetics tools can be used to stimulate gamma motor neurons independently of alpha motor neurons by expressing channelrhodopsin 2 in neuronal PAS domain protein 1‐expressing cells or using lower light intensities with channelrhodopsin 2 expressed in all choline acetyltransferase‐positive motor neurons. This technique could be used to study the role of gamma motor neurons in motor control and proprioception during normal behaviour and disease.

## 1 INTRODUCTION

Two primary types of motor neurons innervate skeletal muscle: the alpha motor neurons, which innervate the contractile extrafusal fibres, and the gamma motor neurons, which innervate the intrafusal fibres of the muscle spindle (Emonet‐Dénand et al., 1975; Kuffler et al., 1951). In addition to different innervation targets, these motor neurons also differ in morphological and electrophysiological characteristics (Kanning et al., 2010). The alpha motor neurons are the most widely studied owing to their important role in voluntary movement. However, gamma motor neurons compose ∼30% of the motor neuron pool and control the length of the sensory region of the muscle spindle, which is an important sensory organ for motor control and the sense of body position in space, or proprioception (Proske & Gandevia, 2012). The muscle spindle is an encapsulated structure composed of specialized bag and chain intrafusal muscle fibres that are innervated by stretch‐sensitive Group Ia and II muscle spindle afferents and gamma motor neurons. Gamma motor neurons form neuromuscular junctions in the polar regions of the intrafusal fibres and control the length of the muscle spindle sensory region and therefore the sensitivity of the muscle spindle afferents (Matthews, 1981). There are many open questions about the function of gamma motor neurons and their role in motor control and disease. For instance, the degree to which gamma motor neurons are activated independently of the alpha motor neurons, especially in humans, is not well understood (Macefield & Knellwolf, 2018). Gamma motor neurons are spared in at least two neuromuscular diseases, amyotrophic lateral sclerosis and spinal muscle atrophy, and spared gamma motor neuron activity might contribute to disease progression (Lalancette‐Hebert et al., 2016; Powis & Gillingwater, 2016). As such, new tools are needed to manipulate gamma motor neuron activity independently of alpha motor neurons to gain a better understanding of fusimotor function.

Electrical stimulation of motor nerves will recruit the largest diameter alpha motor neurons first, and electrical stimulation of gamma motor neuron axons alone requires either teasing apart individual neurons or selective block of alpha motor neurons. In contrast, optical stimulation via the blue light‐activated channelrhodopsin 2 (ChR2) recruits smaller diameter axons first (Arlow et al., 2013). Optical activation has been shown to recruit alpha motor neurons in the more physiologically relevant order of smallest to largest diameter axons (Llewellyn et al., 2010). Given that gamma motor neurons are smaller than the smallest alpha motor neurons in most cases, we hypothesized that they should be the first brought to threshold with optical nerve stimulation. Our first method uses a mouse model that expresses ChR2 in all choline acetyltransferase (ChAT)‐expressing cells, which includes all motor neurons, and uses lower light intensities to activate gamma motor neurons preferentially. Our second method takes advantage of a putative gamma motor neuron marker identified by single‐cell RNA sequencing of motor neurons, neuronal PAS domain protein 1 (Npas1; Blum et al., 2021) to express ChR2 in Npas1‐expressing cells only.

To test the effectiveness of optogenetic motor neuron stimulation, we used an *ex vivo* muscle–nerve preparation, which allowed us to place a light guide directly on the nerve while simultaneously recording muscle spindle afferent firing using an extracellular electrode, in addition to muscle tension with a force transducer (Wilkinson et al., 2012). This provides a functional readout of both alpha and gamma motor neuron activation, with a twitch contraction indicating alpha motor neuron stimulation and increased firing rates of muscle spindle afferents indicating muscle spindle sensory region lengthening via gamma motor neuron stimulation. Here, we describe the results of using optogenetic stimulation to evoke gamma motor neuron firing in mice expressing ChR2 in all ChAT^+^ axons and mice expressing ChR2 in only Npas1‐expressing axons.

## 2 MATERIALS AND METHODS

### 2.1 Ethical approval

All methods and procedures in this study were in accordance with the *Guide for the Care and Use of Laboratory Animals* prepared by the Committee for the Update of the Guide for the Care and Use of Laboratory Animals of the Institute of Laboratory Animal Research, National Research Council (USA) and were approved by the San José State University Institutional Animal Care and Use Committee (protocols 990 and 1047).

### 2.2 Animals

We expressed the blue light‐gated ChR2 in all motor neurons by crossbreeding mice with a floxed ChR2‐enhanced yellow fluorescent protein (EYFP) fusion protein (Ai32[RCL‐ChR2(H134R)/EYFP] or Ai32; RRID:IMSR_JAX:024109; Madisen et al., 2012) with mice expressing Cre recombinase protein in ChR2^+^ neurons (ChAT‐IRES‐Cre; RRID:IMSR_JAX:006410; Rossi et al., 2011). Two male and three female ChAT‐Cre‐ChR2 animals were used for the experiments described.

To express ChR2 in Npas1^+^ cells, we crossed Ai32 mice with mice expressing icre and tdTomato in Npas1‐expressing cells (Npas1‐tdTom; RRID:IMSR_JAX:027718; Hernández et al., 2015; mice a gift from C. Savio Chan). One male and two female Npas1‐Cre‐ChR2 animals were used for the experiments described here.

Mice were housed in cages of 2–10, maintained on a 12 h–12 h light–dark cycle and given ad libitum standard laboratory chow and water. Mice used for experiments were ≥8 weeks of age.

### 2.3 Electrophysiological recording of muscle spindle afferents

**FIGURE 1:**
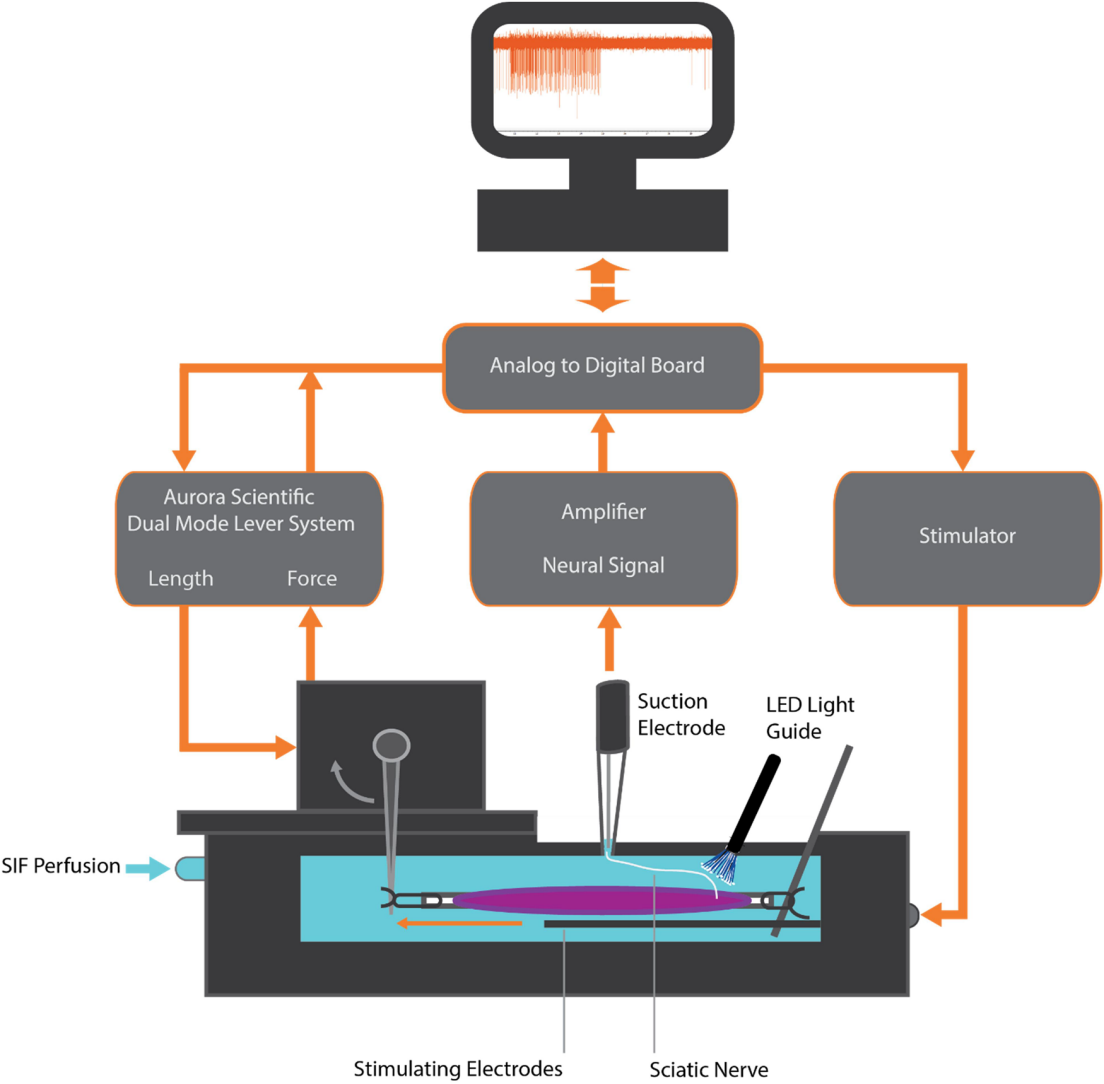
*Ex vivo* muscle–nerve preparation for optogenetic stimulation. The EDL muscle and innervating nerve were placed in an oxygenated tissue bath with SIF. Tendons were sutured to a tissue post and the lever arm of a force and length transducer and controller, which can control muscle length and record muscle force. The peroneal branch of the sciatic nerve was sucked into a glass electrode connected to an extracellular amplifier that recorded muscle spindle afferent firing. The LED light guide was placed on the nerve near the muscle entry point, and blue light (454 nm) of varying optical intensities was used to excite the ChR2‐expressing axons. Figure modified from (Franco et al., 2014). Abbreviations: ChR2, channelrhodopsin 2; EDL, extensor digitorum longus; SIF, synthetic interstitial fluid.

An *ex vivo* muscle–nerve preparation was used to record muscle spindle afferent firing, following the experimental protocol described in more detail previously (Franco et al., 2014; Wilkinson et al., 2012). On the day of experiment, animals were anaesthetized in an induction chamber with inhaled isoflurane (5% isoflurane, 95% O_2_; flow rate 1.5 L/min) until they were insensitive to toe pinch and their breathing had slowed to <20 breaths/min. Animals were then decapitated using sharp scissors and the skinned lower limbs placed in cooled artificial cerebrospinal fluid (mM: 128 NaCl, 1.9 KCl, 1.2 KH_2_PO_4_, 26 NaHCO_3_, 0.85 CaCl_2_, 6.5 MgSO_4_ and 10 glucose, with a pH of 7.4 ± 0.05) and bubbled with 95% O_2_–5% CO_2_. The extensor digitorum longus (EDL) muscle and peroneal branch of the sciatic nerve were dissected and placed in a tissue bath (701C, Aurora Scientific, Inc.; Aurora, ON, Canada) and perfused at a flow rate of 15–30 mL/min with 24°C oxygenated (100% O_2_) synthetic interstitial fluid [mM: 123 NaCl, 3.5 KCl, 0.7 MgSO_4_, 1.7 NaH_2_PO_4_, 2.0 CaCl_2_, 9.5 NaC_6_H_11_O (sodium gluconate), 5.5 glucose, 7.5 sucrose and 10 HEPES, with a pH of 7.4 ± 0.05]. Using 5–0 silk suture, one tendon of the EDL muscle was sutured to a tissue post and the other to a lever arm connected to a force and length transducer (300C‐LR, Aurora Scientific, Inc.; Figure 1).

The EDL muscle was maintained at optimal length (*L*_o_), the length at which maximal twitch contraction is generated following a supramaximal stimulation via bath electrodes (0.5 ms pulse width and supramaximal voltage). The free end of the nerve was suctioned into a glass electrode connected to an extracellular amplifier with head stage (model 1800; A‐M Systems, Sequim, WA, USA) to record neural activity. The recorded neural signal was amplified and digitized using a PowerLab analog‐to‐digital board (AD Instruments; Sydney, NSW, Australia), and LabChart software (ADInstruments) was used to control stimulus timing and record data. A stretch‐sensitive signal was identified as a muscle spindle afferent if it displayed an increase in instantaneous firing frequency and a characteristic slowly adapting response to ramp‐and‐hold stretch, in addition to a pause in firing during twitch contraction. After confirmation of a stretch‐sensitive signal, we passively stretched the muscle (5% *L*_o_; stretch speed 40% *L*_o_/s) to establish the baseline stretch response of our identified muscle spindle afferent.

### 2.4 Optical stimulation

Optical stimulation of ChR2 was delivered using a fibre‐coupled blue LED light (454 nm; M470F3; Thorlabs, Newton, NJ, USA). We placed the ferrule tip of a 400 µm patch cable (Thorlabs) on the nerve near its entry point into the muscle. The location of the patch cable was adjusted until compound action potentials (CAPs) were detected and/or clear increases in muscle spindle afferent firing rates were observed. We then triggered the LED at different optical intensities (1–5 mW) to generate pulse trains (frequencies from 1 to 50 Hz) for 5–60 s each. A duration of 1 min was allowed in between each stimulus train. We chose a pulse width of 2 ms because a previous study (Llewellyn et al., 2010) identified this width to be optimum to elicit a motor neuron response. Testing in our laboratory confirmed that a shorter or longer pulse width of 1 or 20 ms, respectively, was less effective in evoking motor neuron action potentials.

### 2.5 Administration of α‐bungarotoxin

To confirm that light‐evoked changes in muscle spindle afferent firing and muscle contraction were attributable to activation of motor neurons, we used α‐bungarotoxin (BT; Invitrogen B1601) to block neuromuscular junction acetylcholine receptors. Following the optical stimulation protocol, we added 1.25 µM BT to the synthetic interstitial fluid in the tissue bath. We repeated the light stimulation frequency and optical intensity that produced the best modulation of muscle spindle afferent firing pre‐drug every 5 min during a 60 min drug exposure. Light‐evoked firing rates of the muscle spindle afferent were compared pre‐ and post‐drug to determine whether BT could block light‐induced changes in muscle spindle afferent firing. Given that we did not evoke twitch contractions in the Npas1‐Cre‐ChR2 mice, after 60 min we stimulated the nerve at the current intensity required to evoke twitch contraction pre‐drug to confirm extrafusal neuromuscular junction blockade.

### 2.6 Data analysis

We used the spike sorting function in LabChart (AD Instruments) to discriminate the muscle spindle afferent spike shape from the LED‐evoked CAPs. The CAPs were identified as having multiple waveforms within a single stimulus volley owing to the differences in conduction velocities between the excited axons. We then calculated the conduction latency of the fastest volley, which is defined as the time period from the LED stimulation to the peak amplitude of the first wave within the CAP. In one animal where we saw clear evidence of both alpha and gamma motor neuron activation, we did not observe a CAP larger than noise level, potentially owing to the fact that the activated motor neuron axons were not in the sampling area of our electrode. We then calculated the instantaneous firing frequency for the identified muscle spindle afferent and compared before, during and after LED stimulation. To estimate the relative muscle spindle length change in response to gamma motor neuron stimulation in comparison to passive stretch in a given afferent, we also calculated the firing rate of the muscle spindle afferent at the end of a 5% *L*_o_ stretch (3.25–3.75 s into a 4 s ramp‐and‐hold stretch), in addition to the time after a passive stretch or LED stimulation where we observed a pause in muscle spindle afferent firing (pause duration).

## 3 RESULTS

### 3.1 Approach 1: ChAT‐Cre‐ChR2 mice

We first determined whether we could use lower optical intensities in mice with ChR2 expressed in all ChAT^+^ motor neurons to stimulate gamma motor neurons alone.

#### 3.1.1 Slowly conducting gamma motor neurons are recruited at the lowest optical intensities

**FIGURE 2:**
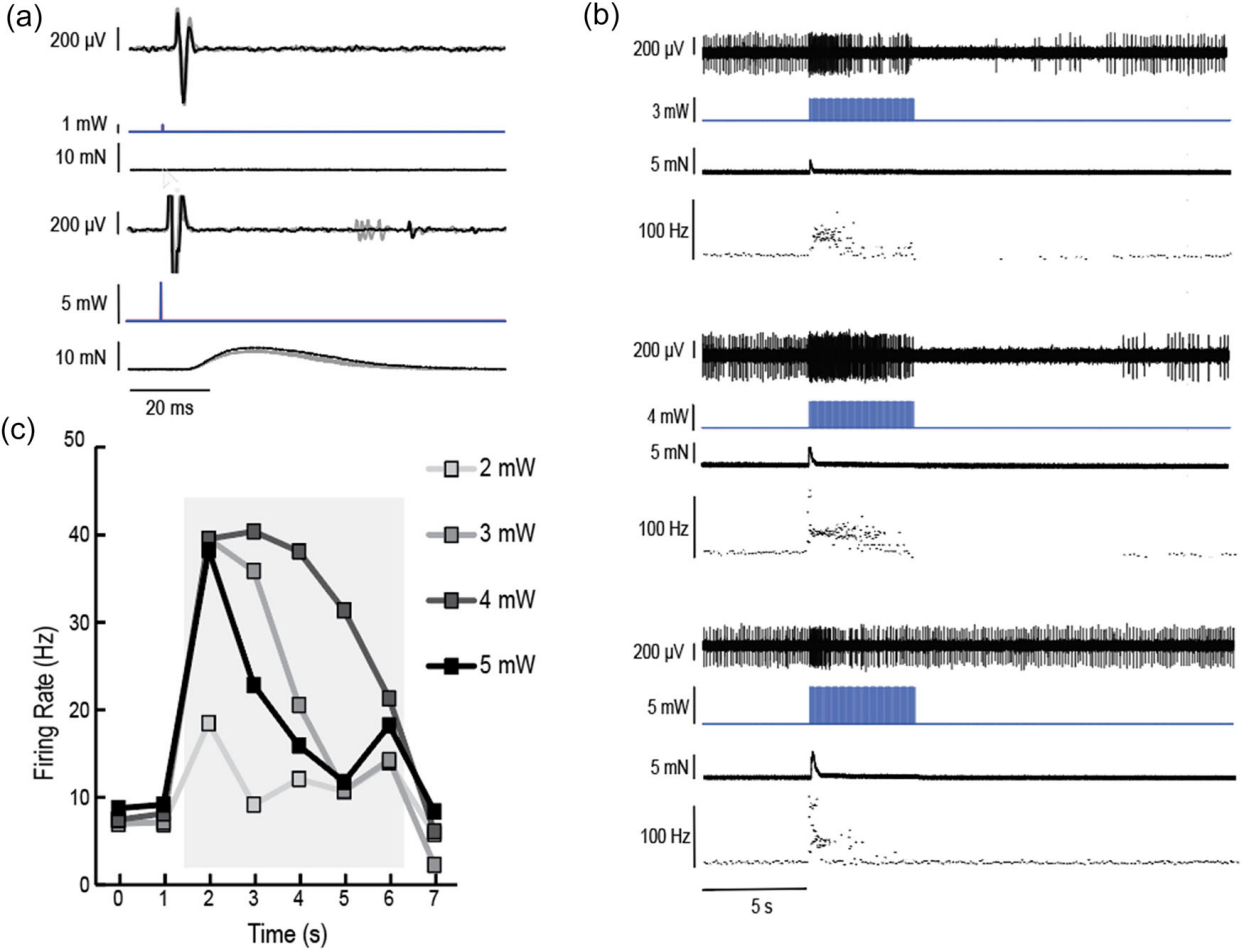
Motor neuron axons are recruited in increasing size as optical intensity increases. (a) At lower light intensities (1 mW; top), the latency of the CAP is longer (∼5 ms), as expected if gamma motor neurons are stimulated. Shorter‐latency CAPs (∼3.6 ms) and twitch contractions are observed at higher light intensities (5 mW; bottom), as expected if alpha motor neurons are recruited. The traces show five stimuli given at 1 Hz overlaid, with the first stimulus in black and subsequent stimuli in grey, from one animal. (b) Optical stimulations for 5 s at 20 Hz (2 ms pulse width) at 3 (top), 4 (middle) and 5 mW (bottom) intensity. In all cases, the raw neural trace of the muscle spindle afferent shown at the top, followed by LED light stimulation (in milliwatts), muscle tension (in millinewtons) and instantaneous firing frequency (in herz) of the afferent. (c) Quantification of muscle spindle afferent firing rates before, during (shaded box) and after LED stimulation at 2–5 mW light intensity. The 4 mW stimulation produced the most sustained increases in afferent firing frequency and produced a prolonged silent period, which is similar to what is observed following passive muscle stretch. Responses in panels b and c were from a single afferent. Abbreviation: CAP, compound action potential.

We confirmed that the smaller diameter gamma motor neurons are recruited at lower optical intensities in the ChAT‐Cre‐ChR2 mice, similar to previous reports showing recruitment of alpha motor neurons from small to large diameter (Llewellyn et al., 2010). We measured the latency of the LED‐evoked back‐propagating CAPs at different optical stimulation intensities while also measuring muscle tension to look for twitch contractions. The CAPs that appeared first at the lowest optical intensities did not produce a twitch contraction and had longer latencies. Higher optical intensities led to shorter latency CAPs that produced a twitch contraction, consistent with alpha motor neuron recruitment (Figure 2a).

Increasing optical intensities at a given frequency of stimulation led to increased twitch contraction strength, as would be expected with increasing alpha motor neuron recruitment (Figure 2b). We also observed increased firing rates of the muscle spindle afferents in response to optical stimulation that were consistent with gamma motor neuron lengthening of the muscle spindle sensory region, in addition to a pause in firing after cessation of the optical stimulation, similar to what is observed after the release of passive stretch (Figure 2b). In general, as optical intensity increased, so did the firing rate and consistency of firing of the muscle spindle afferents. However, at the highest optical intensity (5 mW), firing rates tended to decline, potentially owing to the increased counteraction of muscle spindle lengthening by the alpha motor neuron‐induced contractions and muscle shortening (Figure 2b,c).

#### 3.1.2 Optical stimulation led to frequency‐dependent modulation of muscle spindle afferent firing

**FIGURE 3:**
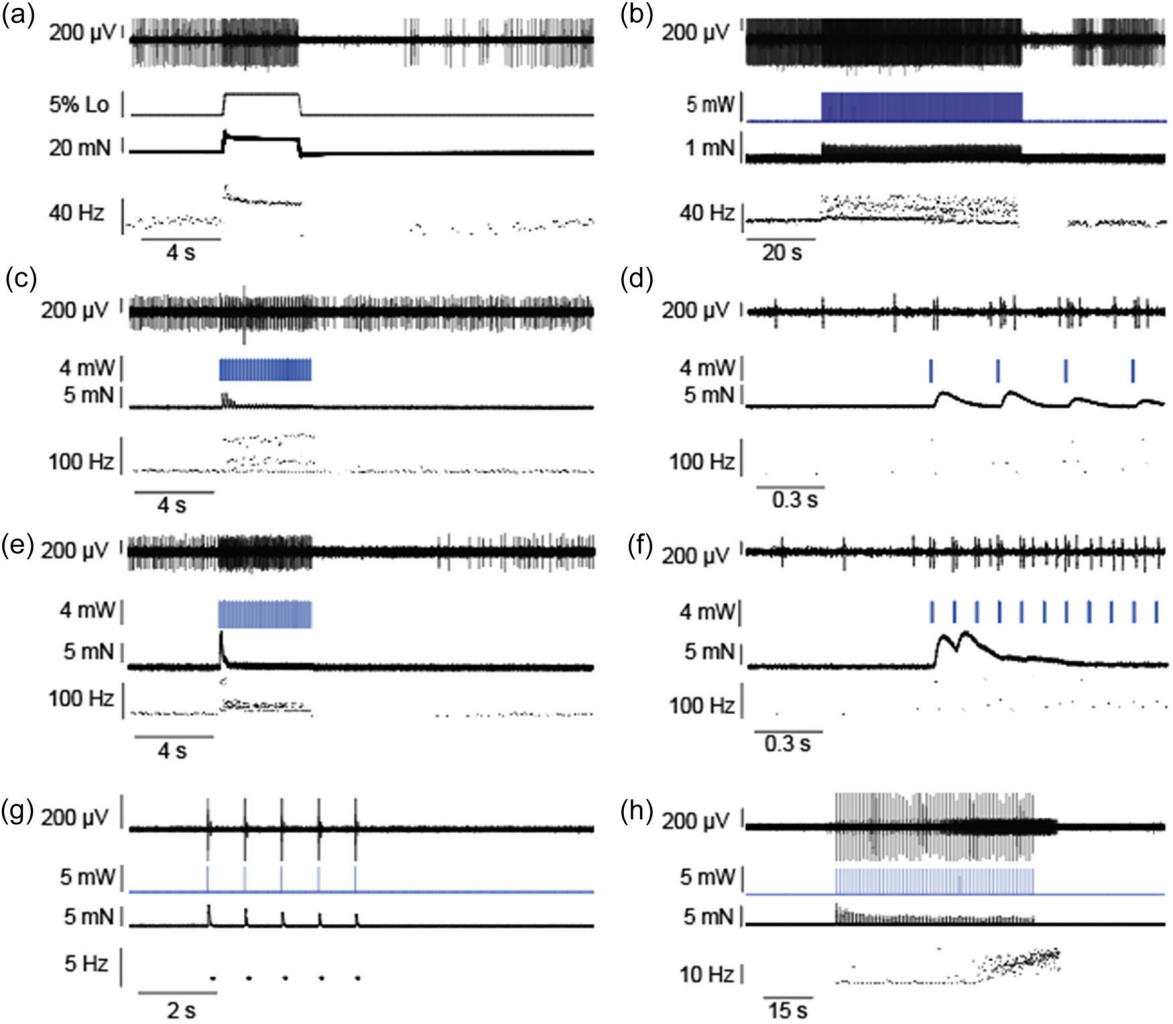
Optical stimulation modulates muscle spindle afferent firing rates. (a) Typical response to a 4 s ramp‐and‐hold passive stretch in a muscle spindle afferent. (b) Response of the same afferent as in panel a in response to a 60 s, 5 Hz LED stimulation (2 ms pulse width), which led to an increase in firing rate and a pause after LED stimulation. (c–f) Another afferent, with similar increases in firing to 5 s, 4 mW light stimulations. (c,d) In response to 5 Hz stimulation, firing rate right after the LED pulse increased to a high level, then paused for an extended period of time (panel d expands the first 0.8 s of stimulation). (e,f) At 15 Hz, a more regular firing frequency was observed after the initial few LED stimulations that also resulted in twitch contractions (panel f expands the first 0.8 s of stimulation). (g,h) Responses to 1 Hz, 5 mW stimulation for 5 or 60 s, respectively, in another afferent that did not have resting discharge. The large spike is the CAP, and the smaller spike is the muscle spindle afferent, which fires at 1 Hz during the 5 s stimulation and the beginning of the 60 s stimulation but increases firing rate to ∼18 Hz near the end of the 60 s stimulation and continues to fire for ∼7 s after light stimulation ends. For panels b–h, the raw neural trace of the muscle spindle afferent shown at the top, followed by LED light stimulation (in milliwatts), muscle tension (in millinewtons) and instantaneous firing frequency (in herz) of the afferent. In panel a, muscle length as a percentage of *L*_o_ is shown as the second trace from the top. Abbreviations: CAP, compound action potential; *L*_o_, optimal length.

Muscle spindle afferents exhibit a characteristic slowly adapting response to passive muscle stretch. They have both static sensitivity, whereby firing rates increase with increasing stretch lengths, and dynamic sensitivity, whereby firing rates are increased more during dynamic stretching and often cease firing as the muscle is shortened (Figure 3a). We observed cessation in firing after optical stimulation in two afferents with resting discharge at both long (60 s; Figure 3b) and shorter optical stimulations (5 s; Figure 3c–f). In one of these afferents, we tested stimulation frequencies of 1–20 Hz at the optimal 4 mW optical intensity as determined above (Figure 2b,c). Increasing stimulation frequencies increased afferent firing rates as would be expected with temporal summation of intrafusal fibre tension. At firing frequencies of ≤10 Hz, the muscle spindle afferent fired at a high rate immediately after the LED stimulation, then exhibited an extended pause before the next afferent action potential (Figure 3c,d). We also observed this pattern during the first few light pulses of 15 Hz stimulation, which were also the only stimulations with appreciable twitch contractions (Figure 3e,f). This pattern of firing could be attributable to dynamic sensitivity of the muscle spindle afferent in response to the muscle shortening via alpha motor neuron activation, which counteracts the gamma motor neuron‐induced lengthening of the muscle spindle sensory region. Alternatively, slower LED stimulation frequencies might also have led to intrafusal fibre lengthening and then shortening, which could have contributed to the dynamic firing observed. At 15 and 20 Hz, we observed a more regular firing rate during optical stimulation, especially after the initial twitch contractions, which is similar to the sustained firing observed during muscle stretch (Figures 2b and 3e,f).

In three afferents that did not have resting discharge, we also observed increased firing with optical stimulation, but there were some differences in the pattern of recruitment in these units. Following many stimulations, especially the shorter (5 s) stimulations, we observed evoked firing solely during light stimulation (Figure 3g). In some cases, especially after longer (60 s) stimulations, we saw prolonged firing even after the LED stimulation ended (Figure 3h). Likewise, we also saw increases in afferent firing rates in the later stages of the 60 s stimulation in two afferents (Figure 3h; other afferent not shown). Future studies are needed to determine the precise cause of this continued firing after the cessation of gamma motor neuron stimulation.

Although lower light intensities preferentially recruited more slowly conducting, putative gamma motor neurons, in all five animals tested we still observed twitch contractions at least during the beginning of stimulation (Figure 3). In some animals, the evoked twitch contractions were very small (<1 mN), but these results suggest that varying light intensity alone will not eliminate all alpha motor neuron stimulation using this approach.

### 3.2 Approach 2: Npas1‐Cre‐ChR2 mice

Owing to the inability to separate alpha and gamma motor neuron stimulation strictly in the ChAT‐Cre‐ChR2 mice, we next tested whether we could express ChR2 in gamma motor neurons alone using a recently identified putative specifically gamma motor neuron‐expressed transcription factor, Npas1 (Blum et al., 2021).

#### 3.2.1 Expressing ChR2 in Npas1^+^ cells allows for preferential stimulation of gamma motor neurons

**FIGURE 4:**
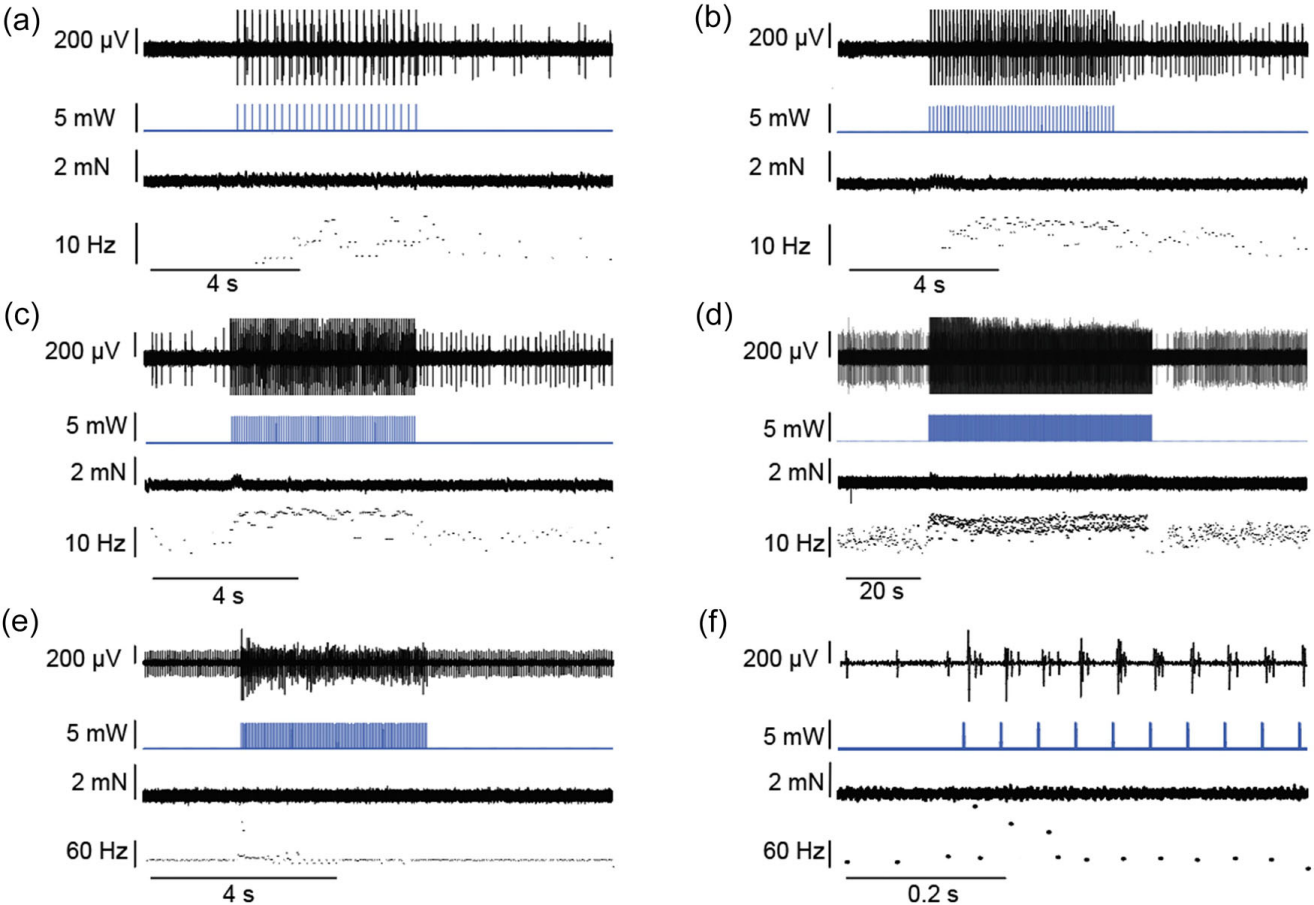
Optical stimulation modulates muscle spindle afferent firing rates in Npas1‐Cre‐ChR2 afferents. Response to 5 s LED stimulations (2 ms pulse width) at 5 (a), 10 (b) and 15 Hz (c). (d) Response to a 60 s, 15 Hz LED stimulation in the same afferent. (e) Response to a 5 s, 20 Hz stimulation in another afferent with resting discharge (panel f shows expanded first 0.8 s of stimulation). The CAP amplitude in this unit decreased quickly and indicates decreased axonal recruitment over time that explains the lack of sustained LED‐evoked afferent firing. For all panels, the raw neural trace of the muscle spindle afferent shown at the top, followed by LED light stimulation (in milliwatts), muscle tension (in millinewtons) and instantaneous firing frequency (in herz) of the afferent. Abbreviations: CAP, compound action potential; ChR2, channelrhodopsin 2; Npas1, neuronal PAS domain protein 1.

Similar to what was observed in the ChAT‐Cre‐ChR2 mice, light stimulation in Npas1‐Cre‐ChR2 mice led to increased muscle spindle afferent firing in three animals (Figure 4). In one afferent that did not have resting discharge, as LED stimulation frequency increased from 5 to 15 Hz, firing rates of the muscle spindle afferent also increased (Figure 4a–c). At 15 Hz stimulation, firing rates reached ∼14 Hz during light stimulation, which was very close to the level of firing observed in this afferent at the end of a 5% *L*_o_ passive stretch (18 Hz). We also observed firing after the cessation of LED stimulation that increased in frequency as LED stimulation frequency increased (Figure 4a–c). This firing was maintained after a 5 s, 15 Hz stimulation for >1 min. When a 60 s, 15 Hz stimulation was then given, we saw maintained afferent firing for the duration of the light stimulation followed by a pause in firing (Figure 4d) that was similar to what we observed in ChAT‐Cre‐ChR2 afferents with resting discharge (Figure 2b,e) or in response to passive stretch (Figure 2a). In two of the tested afferents, LED‐induced muscle spindle afferent firing modulation was observed only during the beginning of the light stimulation (Figure 4e,f). In these animals, CAP amplitude decreased rapidly after the first few LED stimulations, suggesting decreased gamma motor neuron recruitment that would explain the inability to maintain LED‐evoked afferent firing. The use of a higher‐powered laser and/or closer light guide placement could potentially improve the stability of axonal recruitment in the future.

We also wanted to confirm a lack of alpha motor neuron stimulation in these animals and looked for light‐evoked twitch contractions. In two of the three animals, we saw no evidence of twitch contractions (Figure 4e,f). In one animal, we did see what could be considered twitch contractions, but they were very small in amplitude (< 0.5 mN; Figure 4a–d). If these were alpha motor neuron‐mediated twitch contractions, they were much smaller than anything observed in the ChAT‐Cre‐ChR2 animals.

#### 3.2.2 Neuromuscular junction blockade eliminated light‐activated increases in muscle spindle afferent firing

To ensure that the LED‐evoked muscle spindle afferent firing observed was attributable to gamma motor neuron‐mediated muscle spindle lengthening and not direct recruitment of muscle spindle afferents, we blocked the nicotinic acetylcholine receptors of the neuromuscular junction with α‐bungarotoxin (1.25 µM BT) and recorded the response to LED stimulation for 1 h in the afferent depicted in Figure 4a–d. We observed a progressive decrease in light‐evoked firing in the tested afferent that was completely eliminated by 50 min of BT exposure. We confirmed that all neuromuscular junctions were blocked by showing that a stimulating electrode placed on the nerve was unable to elicit twitch contraction. We also confirmed that the recorded afferent still had regular firing in response to a 5% *L*_o_ stretch. After 50 min of BT, firing rates at the end of the stretch were the same as that recorded immediately before adding BT (18 Hz). These results support the hypothesis that the optical stimulus is recruiting gamma motor neurons, which lengthen the muscle spindle sensory region to increase muscle spindle afferent firing.

#### 3.2.3 Recruitment of motor neuron axons was not maintained throughout optical stimulation, especially at higher stimulation frequencies

Our tests revealed that the same level of motor neuron axon recruitment was not maintained throughout optical stimulation in both ChAT‐ChR2‐Cre and Npas1‐ChR2‐Cre animals (data quantified for four total animals), especially of larger diameter axons and at higher stimulation frequencies. We observed lower motor neuron recruitment of faster conducting axons as measured by CAP latency increases over a 5 s optical stimulation period, especially at frequencies of ≥20 Hz (Table 1; quantification from three other animals similar but not shown). This suggests some level of reduced axonal recruitment over time. It also matches observations that at some optical intensities twitch contractions would be present only at low frequencies (1–5 Hz) and at the beginning of a higher frequency stimulation at a given optical intensity (Figure 3c–f). Reproducible recruitment throughout light stimulation might require a higher‐powered laser and/or light‐activated ion channels with faster kinetics.

**TABLE 1:** Compound action potential (in milliseconds) increases with lower optical intensity and over the 5 s stimulation.

5 mW	1 Hz	10 Hz	20 Hz	30 Hz	40 Hz	50 Hz
First	3.5	3.7	3.5	3.6	3.7	3.6
Middle	3.8	4.4	4.6	5.1	6.2	–
Last	3.8	4.4	4.6	5.2	6.1	–
						
**3 mW**	**1** **Hz**	**10** **Hz**	**20** **Hz**	**30** **Hz**	**40** **Hz**	**50** **Hz**
First	4.5	4.2	4.2	4.2	4.1	4.2
Middle	4.5	5	5.3	5.8	6.3	–
Last	4.4	4.9	5.5	6.7	–	–

*Note*: Time from the LED stimulation to the first peak deflection of the CAP, or latency (in milliseconds), at the first, middle and last optical stimulation from a 5 s stimulus of the frequency listed at the top of the column for either 5 or 3 mW optical stimulation intensity. Data are from one representative experiment, although quantification from three other experiments over a smaller frequency range exhibited similar trends, as shown in the Supplementary Material.

## 4 DISCUSSION

Here, we describe two different optogenetic mouse models that can be used to stimulate gamma motor neurons and lead to physiologically relevant increases in muscle spindle afferent firing rates. The most promising approach for specific gamma motor neuron stimulation uses a newly identified marker that appears to be present in gamma but not alpha motor neurons, Npas1 (Blum et al., 2021). We confirmed that optical stimulation of the sciatic nerve in mice where Npas1^+^ cells express ChR2 could lead to increases in muscle spindle afferent firing rates (Figure 3). As expected, higher LED stimulation frequencies led to increased muscle spindle afferent firing rates consistent with larger and/or more prolonged lengthening of the muscle spindle sensory region. LED stimulation led to only very small twitch contractions, which could suggest some ChR2 expression in alpha motor neurons or in beta motor neurons, which innervate both extrafusal and intrafusal fibres (Bessou et al., 1965). However, the contractions were much smaller than what was observed in ChAT‐Cre‐ChR2 mice, suggesting better gamma motor neuron specificity.

**FIGURE 5:**
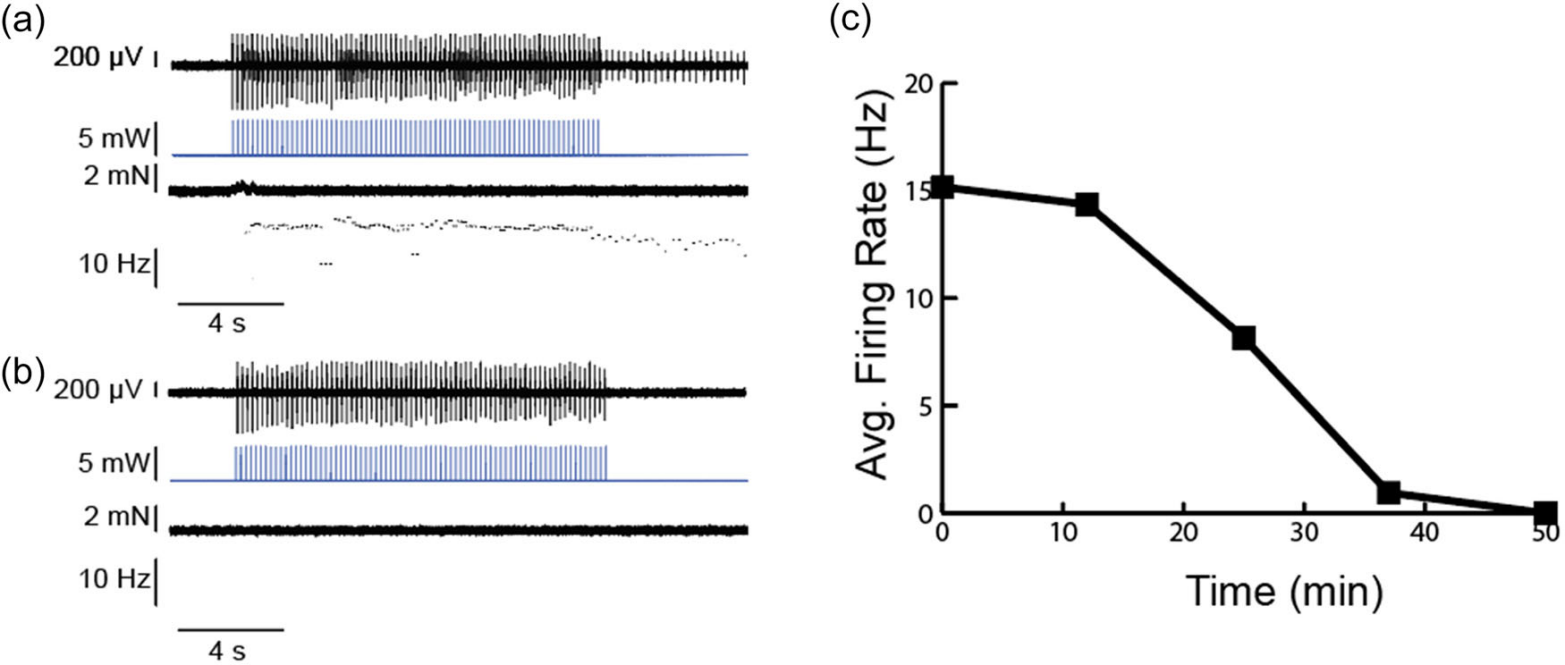
Neuromuscular junction blockade blocks light‐evoked muscle spindle afferent responses. (a,b) Response of one afferent to 5 s LED stimulations (2 ms pulse width) at 15 Hz before (a) and 50 min after administration of α‐bungarotoxin, where all light‐evoked firing is eliminated (b). (c) Quantification of average muscle spindle afferent firing rate during the 5 s LED stimulation before (0 min) and up to 50 min after α‐bungarotoxin. For panels a and b, the raw neural trace of the muscle spindle afferent shown at the top, followed by LED light stimulation (in milliwatts), muscle tension (in millnewtons) and instantaneous firing frequency (in herz) of the afferent.

Npas1 is a basic helix–loop–helix–PAS transcription factor found in brain and spinal cord tissues (Zhou et al., 1997). RNA sequencing studies have not shown appreciable levels of Npas1 in proprioceptor cell bodies (Oliver et al., 2021; Zheng et al., 2019), hence we think it unlikely that ChR2 is expressed in muscle spindle afferents. However, we confirmed that the observed LED‐induced muscle spindle afferent firing was attributable to gamma motor neuron‐mediated intrafusal fibre contraction by blocking the light‐induced increased firing with the neuromuscular junction blocker, α‐bungarotoxin (Figure 5). This proof‐of‐concept study also supports the use of Npas1‐Cre‐driven expression of other targeted proteins for the manipulation and/or imaging of gamma motor neurons.

We also expanded on previous work using ChAT‐Cre‐ChR2 mice to show that the optical recruitment of motor axons is from small diameter to large (Llewellyn et al., 2010). As expected, lower optical intensities primarily recruited the smaller gamma motor neurons. We observed slower latencies of back‐propagating CAPs with lower optical intensities and that higher optical intensities were needed to cause alpha motor neuron‐induced twitch contractions (Figure 2). LED stimulation increased firing of muscle spindle afferents that was modulated by stimulation frequency as predicted (Figure 3). Although we could obtain recruitment at the lower optical intensities consistent with primary recruitment of gamma motor neurons, we were never able to eliminate alpha motor neuron recruitment completely and still obtain high levels of muscle spindle afferent modulation. This limits the use of these mice for studies where it is important to stimulate only gamma motor neurons. ChAT‐Cre‐ChR2 mice would be useful for studies interested in co‐activating alpha and gamma motor neurons, and we note that previous work using these mice to target alpha motor neurons would have also recruited gamma motor neurons and probably changed muscle spindle afferent firing rates. ChAT has been found in muscle spindle afferents (Zhang et al., 2014); therefore, our light stimulation might also have directly stimulated the muscle spindle afferents themselves. Group Ia and II muscle spindle afferent diameters and conduction velocities are similar to those of alpha motor neurons, hence we expect that higher optical intensities were also necessary to recruit their axons. Given the increased muscle spindle afferent firing rates seen after the time period that direct recruitment would occur and the induced pause in firing after light stimulation ended that we observed, we feel it is unlikely that our results were solely attributable to the presence of ChR2 in muscle spindle afferents.

Given that we are using light shone onto a whole nerve and not teased afferents, we think it likely that we are recruiting multiple gamma motor neurons but will only be assaying the function of those innervating the same muscle spindle as the afferent we are recording. There are two types of gamma motor neurons, the static gamma motor neurons, which innervate the Bag 2 and nuclear chain intrafusal fibres, and the dynamic gamma motor neurons innervating the Bag 1 fibres (Matthews, 1981). In the cat, both static and dynamic gamma motor neurons are reported to have similar axon diameters and conduction velocities (Barker et al., 1973; Rossi‐Durand, 2006), suggesting that optical stimulation should recruit these subtypes at similar thresholds. Increases in gamma motor neuron firing during rest that lead to increased muscle spindle afferent firing will be caused by static gamma motor neurons. Dynamic gamma motor neuron stimulation will increase dynamic sensitivity of only Group Ia afferents (Matthews, 1981). We focused on assaying primarily static gamma motor neuron function, because all muscle spindle afferents should be responsive to that stimulation. Additionally, given the presence of back‐propagating CAPs, which will drown out muscle spindle afferent firing for a window of time, minor changes in dynamic firing rates are difficult to measure in our present set‐up. Future studies should attempt to separate static and dynamic gamma motor neuron stimulation, by finding either uniquely expressed proteins or other ways to drive a specific type of gamma motor neuron or by using teased axons to allow for recording over the entire stimulation time.

### 4.1 Limitations

We were not able to drive gamma motor neurons reliably much above 20 Hz with light stimulation and saw decreased axonal recruitment over even short optical stimulations that seemed to affect the larger axons preferentially (Table 1). Previous in vivo optical stimulation studies of motor axons have found decreased effectiveness of stimulation rates of >36 Hz (Towne et al., 2013). Therefore, we might expect that increasing the bath temperature to body temperature will allow axons to follow at slightly higher stimulation frequencies. However, gamma motor neurons have higher firing rates than alpha motor neurons, with firing rates of ≤100 Hz observed in decerebrate cats (Hunt, 1951). The inability to drive axonal firing rates to higher frequencies using optical stimulation is probably attributable to inactivation of ChR2 and might be solved by using optogenetic channels with faster kinetics, such as the E123T mutation in ChR2 (ChETA) (Gunaydin et al., 2010; Towne et al., 2013).

More concerningly, there are reports that the immune system targets the ChR2 protein, leading to axonal blebbing and cytotoxicity (Maimon et al., 2018). Others have shown age‐dependent declines in the effectiveness of optical stimulation of motor axons in multiple species consistent with cytotoxicity (Maimon et al., 2017; Williams et al., 2019). Recording from animals at earlier time points or using acute virally mediated expression of ChR2 might minimize any immune system‐mediated cytotoxicity.

### 4.2 Future directions

We propose that optogenetic stimulation or expression of other proteins, such as the designer receptors exclusively activated by designer drugs (DREADDs), especially using the gamma motor neuron‐specific Npas1‐Cre‐ChR2 mice, can provide a useful way to control gamma motor neuron activity to probe the role of the fusimotor system in motor control and disease. For instance, this method could be used to screen for intrafusal fibre and/or gamma motor neuron dysfunction in response to drugs added to the ex vivo bath, which could be useful in screening for potential side effects on the muscle spindle. Modifying the technique to allow for real‐time imaging of individual muscle spindles could also provide important information about intrafusal fibre contraction dynamics and alterations in response to developmental stage, disease or drug addiction. Many diseases, for instance Amish nemaline myopathy (Oki et al., 2019), are caused by mutations to proteins found in both extrafusal and intrafusal fibres. Our technique could potentially be used to determine whether these diseases lead to changes in muscle spindle afferent responsiveness to gamma motor neuron stimulation.

Controlling gamma motor neuron firing during motor behaviours in freely behaving animals would provide important insight into the role of the fusimotor system in motor control. Others have used similar optogenetic techniques to stimulate motor axons in vivo in rodents (Maimon et al., 2018; Matarazzo et al., 2023; Towne et al., 2013; Ward et al., 2016) and even a non‐human primate model (Williams et al., 2019). Similar to what we observed, these studies were limited by the ability to stimulate the axons at only relatively slow frequencies. Some studies also report age‐dependent decreases in stimulation effectiveness and potential cytotoxicity (Maimon et al., 2018, 2017; Williams et al., 2019). Some of these problems might be addressed by using optogenetic proteins with faster kinetics, expressing the optogenetic protein later in development and/or developing new optogenetic proteins that do not cause cytotoxicity. Improved nerve cuffs, potentially using a combination of optical stimulation and subthreshold electrical stimulation, might also improve stimulation efficacy (Matarazzo et al., 2023).

## 5 CONCLUSION

In summary, we have shown that optical stimulation of ChR2 in gamma motor neurons can cause physiologically relevant changes in muscle spindle length that can be assayed by recording muscle spindle afferent firing and propose that this method can be used to study fusimotor function.

## AUTHOR CONTRIBUTIONS

All experiments were conducted at San José State University in the laboratory of Katherine A. Wilkinson. Katherine A. Wilkinson and Shawn Hochman contributed to conceptualization and design. Apoorva Karekal, Remie Mandawe, Cameron Chun, Sai Kiran Byri, Danitza Cheline and Serena Ortiz were responsible for data collection. Apoorva Karekal, Remie Mandawe, Cameron Chun, Sai Kiran Byri and Katherine A. Wilkinson contributed to data analysis. Katherine A. Wilkinson was responsible for drafting the article, and all authors reviewed the article and provided critical feedback. Apoorva Karekal and Remie Mandawe contributed equally to this work. All authors approved the final version of the manuscript and agree to be accountable for all aspects of the work in ensuring that questions related to the accuracy or integrity of any part of the work are appropriately investigated and resolved. All persons designated as authors qualify for authorship, and all those who qualify for authorship are listed.

## CONFLICT OF INTEREST

None declared.

## Supporting information



Supporting Information

## Data Availability

Quantified firing rates of afferents shown in the figures are available in a supplementary data file in this article. Raw data files are available from the corresponding author upon reasonable request.
